# Comorbidities in early-onset sporadic versus presenilin-1 mutation-associated Alzheimer disease dementia: Evidence for dependency on Alzheimer disease neuropathological changes

**DOI:** 10.1093/jnen/nlae122

**Published:** 2024-12-04

**Authors:** Diego Sepulveda-Falla, Carlos Andrés Villegas Lanau, Charles White III, Geidy E Serrano, Juliana Acosta-Uribe, Barbara Mejía-Cupajita, Nelson David Villalba-Moreno, Pinzhang Lu, Markus Glatzel, Julia K Kofler, Bernardino Ghetti, Matthew P Frosch, Francisco Lopera Restrepo, Kenneth S Kosik, Thomas G Beach

**Affiliations:** Institute of Neuropathology, University Medical Center Hamburg-Eppendorf, Hamburg, Germany; Faculty of Medicine, Neuroscience Group of Antioquia, University of Antioquia, Medellin, Colombia; Neuropathology Section, Department of Pathology, University of Texas Southwestern Medical Center, Dallas, TX, United States; Civin Laboratory for Neuropathology, Banner Sun Health Research Institute, Sun City, AZ, United States; Faculty of Medicine, Neuroscience Group of Antioquia, University of Antioquia, Medellin, Colombia; Neuroscience Research Institute, University of California Santa Barbara, Santa Barbara, CA, United States; Department of Molecular, Cellular, and Developmental Biology, University of California Santa Barbara, Santa Barbara, CA, United States; Faculty of Medicine, Neuroscience Group of Antioquia, University of Antioquia, Medellin, Colombia; Neuroscience Research Institute, University of California Santa Barbara, Santa Barbara, CA, United States; Department of Molecular, Cellular, and Developmental Biology, University of California Santa Barbara, Santa Barbara, CA, United States; Institute of Neuropathology, University Medical Center Hamburg-Eppendorf, Hamburg, Germany; Institute of Neuropathology, University Medical Center Hamburg-Eppendorf, Hamburg, Germany; Institute of Neuropathology, University Medical Center Hamburg-Eppendorf, Hamburg, Germany; Department of Pathology, University of Pittsburgh, Pittsburgh, PA, United States; Department of Pathology and Laboratory Medicine, Indiana University School of Medicine, Indianapolis, IN, United States; Department of Pathology, Massachusetts General Hospital and Harvard Medical School, Boston, MA, United States; Faculty of Medicine, Neuroscience Group of Antioquia, University of Antioquia, Medellin, Colombia; Neuroscience Research Institute, University of California Santa Barbara, Santa Barbara, CA, United States; Department of Molecular, Cellular, and Developmental Biology, University of California Santa Barbara, Santa Barbara, CA, United States; Civin Laboratory for Neuropathology, Banner Sun Health Research Institute, Sun City, AZ, United States

**Keywords:** apolipoprotein E, autopsy, cerebral infarcts, clinical trial, E280A, Lewy body, TDP-43, white matter rarefaction

## Abstract

Studying comorbidities in early onset Alzheimer disease (AD) may provide an advantageous perspective on their pathogenesis because aging factors may be largely inoperative for these subjects. We compared AD comorbidities between early-onset sporadic cases and American and Colombian cases with *PSEN1* mutations. AD neuropathological changes (ADNC) were very severe in all groups but more severe in the *PSEN1* groups. Lewy body disease and cerebral white matter rarefaction were the most common (up to 60%) of AD comorbidities, followed by arteriolosclerosis (up to 37%), and large-vessel atherosclerosis (up to 20%). Differences between the 3 groups included earlier age of onset in the American *PSEN1* cases, shorter disease duration in sporadic cases, and more frequent large-vessel atherosclerosis and cerebral amyloid angiopathy in the Colombian *PSEN1* cases. Logistic regression models adjusted for age and sex found the presence of a *PSEN1* mutation, an apolipoprotein ε4 allele and TDP-43 pathology to predict an earlier age of onset; Hispanic ethnicity and multiracial subjects were predictive of severe CAA. Comorbidities are common in early onset AD and should be considered when planning clinical trials with such subjects. However, they may be at least partially dependent on ADNC and thus potentially addressable by anti-amyloid or and/anti-tau therapies.

## INTRODUCTION

Autopsy studies have demonstrated that comorbid neurodegenerative and cerebrovascular disease occur in the great majority of subjects with Alzheimer disease (AD) and are likely to additively alter the rate of decline or severity of cognitive impairment.^1–5^ Some of these AD comorbidities, including Lewy body disease (LBD), hippocampal sclerosis, TDP-43 proteinopathy, and cerebral infarcts, can cause dementia even when AD neuropathological changes (ADNC) are low or absent. Comorbidities may interfere with the evaluation of AD clinical trials as the subjects may not respond to ADNC-specific molecular therapeutics. At present, it is not possible to stratify AD trials for the presence of comorbidity as there are no proven sensitive and specific diagnostics or disease-modifying therapies. It is possible, however, that at least some comorbidities may to some degree be secondary consequences of ADNC. If this were true, then effective ADNC-specific therapeutics might also reduce the extent or severity of comorbid pathologies. Because it has often been assumed that these comorbidities would be largely absent in younger, clinical trial-aged sporadic AD subjects, most reports have focused on the very old because comorbidities increase with age. However, it has recently been shown that comorbidities, particularly LBD, white matter rarefaction (WMR), and TDP-43 proteinopathy, are present even in many cases of sporadic early-onset AD (sEOAD).^6^^,^^7^

Much less is known about the prevalence of comorbidities in genetically determined early-onset AD caused by autosomal dominant mutations in genes such as presenilin-1 (*PSEN1*). This is a critical knowledge gap as AD in this setting may provide an advantageous perspective on the role of aging in the pathogenesis of AD comorbidities as aging factors may be largely inoperative for these subjects. Additionally, as subjects with both sEOAD and genetic EOAD are increasingly entered into clinical trials, it is important to understand their comorbidity rates so that subject stratification might allow the recognition of significant trial outcomes for subject subsets, even when overall results are negative. Because AD associated with *PSEN1* mutations has a presumed single-cause etiology and the average age at death is under 60 years, any comorbidities in this setting may be considered as at least partially secondary to the causative AD mechanisms rather than aging, and thus indicate whether effective AD therapeutics may also be effective for comorbidities.

In this study, we sought to compare the prevalences and types of AD comorbidities between subjects with sEOAD vs AD associated with *PSEN1* mutations, the most common cause of early-onset autosomal dominant AD. In particular, we were able to ascertain, for the first time, the prevalences of a fairly complete set of AD comorbidities in the United States *PSEN1* cases as well as in the Colombian *PSEN1* E280A kindred.^8^^,^^9^ A previous study used NACC data to profile the neuropathology of *PSEN1* mutation AD,^10^ but less comorbidity data were routinely reported at that time.

## METHODS

The approach used in this study is the same as used in a previous survey of neuropathological comorbidities in sporadic AD using data from the National Alzheimer Coordinating Center (NACC).^6^^,^^11^^,^^12^ Included subject data for AD in the present study were restricted to those that had dementia and met NIA-AA intermediate or high ADNC criteria^13^^,^^14^; the data were subdivided by the presence or absence of a *PSEN1* mutation. All other dementia-associated mutation types were excluded and those without a known dementia-associated mutation were restricted to those who died under the age of 60 years. Only data from NACC Neuropathology Form 10 were used (https://files.alz.washington.edu/documentation/rdd-np.pdf), as this is the largest dataset that is most inclusive of comorbidity data. Also included were comparative data for *PSEN1* AD subjects from the US, derived from the same NACC dataset and from Colombia. The United States cases included subjects with several different *PSEN1* mutations whereas the Colombian cases all had the E280A mutation^8^^,^^9^; 36 of the latter have been reported in earlier neuropathological studies.^15^^,^^16^

Pathology categories investigated included the major AD-specific lesions: senile or amyloid plaques (NACC variable NPTHAL, all plaque types classified by Thal amyloid phase^17^), neurofibrillary tangles (NACC variable NPBRAAK, classified by Braak stage^18^^,^^19^), neuritic plaques (NACC variable NPNEUR, densities classified according to CERAD),^20^ diffuse plaques (NACC variable NPDIFF, classified analogously to CERAD neuritic plaques), amyloid angiopathy (NPAMY; classified as none, mild, moderate, or severe), and NIA-AA AD Neuropathological Change Level (NACC variable NPADNC^13^^,^^14^).

Comorbid neurodegenerative conditions investigated included LBD; NACC variable NPLBOD, with regional data for olfactory bulb-only (LB-OB), brainstem predominant (LB-BS), amygdala-predominant (LB-Amyg), limbic (LB-Limb), and neocortical (LB-Neo) stages. TDP-43 proteinopathy (TDPNOS) was recorded as present or absent based on combined results with NACC variables NPTDPA, B, C, D, and E (for spinal cord, amygdala, entorhinal area, hippocampus, and neocortex, respectively). Non-AD tauopathy was recorded as NACC variable NPFTDTAU, for any non-AD tauopathy including progressive supranuclear palsy, corticobasal degeneration, Pick disease, argyrophilic grains, chronic traumatic encephalopathy, or “other”. For each comorbid pathology and AD group, the proportion of cases possessing that pathology was determined.

Comorbid cerebrovascular conditions investigated included circle of Willis arteriosclerosis (NACC variable NACCAVAS; for this study only “moderate” and “severe” qualified for the presence of the condition), old gross cerebral infarcts (NACC variable NPINF; for this study, 1 or more large or lacunar infarcts qualified for the condition), old microscopic infarcts (NACC variable NPOLD; for this study, 1 or more old microinfarcts qualified for the condition), old microscopic hemorrhages (NACC variable NPOLDD; for this study, 1 or more old microhemorrhages qualified for the condition), arteriolosclerosis (NACC variable NACCARTE; for this study only “moderate” and “severe” qualified for the presence of the condition), and WMR (NACC variable NPWMR; for this study only “moderate” and “severe” qualified for the presence of the condition). For each comorbid pathology, the proportion of cases possessing that pathology, relative to those for which the absence of the pathology was specifically recorded, was determined.

Statistical methods used for continuous data included analysis of variance (ANOVA) with post hoc paired comparisons by the Tukey test. Chi-square and Fisher exact tests were used for comparisons of proportional data. For all tests, the significance level was set at *P *< .05. Logistic regression models were used to determine the independent strength and significance of several variables for earlier onset age, shorter disease duration, circle of Willis atherosclerosis severity, and amyloid angiopathy severity. The independent variables for these models included age, sex, the *APOE-*ε4 allele, race, and Hispanic vs non-Hispanic ethnicity.

## RESULTS

Demographic and mutation data for all cases are shown in Table 1. As a result of our selection criteria, all sEOAD cases were less than 60 years old at death, with a mean age of 56.3 years; of the 33 cases, 15 had not had genetic testing done to confirm the absence of a causative gene mutation in *APP*, *PSEN1*, *PSEN2*, or *c9orf72*, however, a separate comparison of test-confirmed and unconfirmed cases found no significant differences (Supplementary Tables) and so all cases were included in subsequent analyses. The United States *PSEN1* cases were younger at symptom onset and death than the other groups. The sEOAD cases were all non-Hispanic and Caucasian/White and had the shortest disease durations. The United States *PSEN1* subjects were all Caucasian/White; 13 were non-Hispanic while 6 were Hispanic. There were 12 different *PSEN1* mutations in the United States cases. All Colombian cases were multiracial-Hispanic and had the *PSEN1* E280A mutation.

**Table 1.** Demographics and mutation genotypes for *PSEN1* and sporadic early-onset AD (sEOAD) cases.

Group	Age at onset^a^(mean, SD, range)	A. Age at death^b^ B. Disease Duration^c^ (mean, SD, range)	Sex	*PSEN1* mutation genotypes	
				Mutation	*N* (cases)
***PSEN1*—United States** ***N* = 19**	42.81; 6.66; 33-58	A. 53.4; 8.6; 34-75 B. 11.6; 5.12; 603	6 M, 13F	A431E	5
				G206A	3
				A426P	2
				P88L	2
				H163R	2
				P267A	1
				G206E	1
				M233L	1
				N135S	1
				S130L	1
***PSEN1*—Colombia** ***N* = 50**	47.8; 5.32; 37-62	A.57.7; 7.25; 42-79 B. 9.88; 4.52; 2-22	15M, 35F	E280A (All)	
**sEOAD—United States** ***N* = 33**	48.3; 2.84; 41-54	A. 56.3; 2.98; 47-59 B. 4.0; 1.87; 1-10	25M; 8 F	N/A	

Abbreviations: *PSEN1*=presenilin-1; sEOAD=sporadic early-onset Alzheimer disease.

a ANOVA *P* = .003; Tukey *P* < .01 for *PSEN1—United States* vs all other groups.

b ANOVA *P* = .075; Tukey *P* < .05 for *PSEN1—United States* vs *PSEN1—COL*.

c ANOVA *P* < .0001; Tukey *P* < .001 for *PSEN1 groups vs SEOAD*.

Confirmatory of earlier reports,^6–8^^,^^10^ ADNC is consistently very severe in early-onset cases, whether they are sporadic or autosomal dominant (Table 2). All 3 groups had median scores that were the highest possible for Thal amyloid phase, Braak neurofibrillary stage, diffuse plaque density, and NIA-AA ADNC Level. When the mean scores were compared, however, ANOVA showed significant group differences for Thal amyloid phase, diffuse plaque density, and ADNC Level; on pairwise comparisons for these, *PSEN1* groups had significantly greater scores than the sEOAD group. ANOVA and pairwise comparisons for CERAD neuritic plaque density and Braak neurofibrillary stage, however, were not significant.

**Table 2.** Autopsy data for AD-related neuropathological variables in sEOAD and *PSEN1* AD cases.

Group	ApoE-4	ApoE-2	**NPTHAL** ^a^	NPBRAAK	NPNEUR	**NPDIFF** ^b^	**NPADNC** ^c^	**NPAMY** ^d^
***PSEN1*—United States**	6/14	2/14	5; 5	6; 6	3; 3	3; 3	3; 3	2.10; 2
	42.9%	14.3%						
***PSEN1*—COL**	9/34	1/34	5; 5	6; 6	2.9; 3	3; 3	3; 3	2.58; 3
	26.5%	2.94%				(*n* = 36)		
**sEOAD**	16/33	2/33	4.72; 5	5.79; 6	2.85; 3	2.70; 3	2.88; 3	1.42; 1
	48.5%	6.1%						

Abbreviations: *PSEN1*=presenilin-1; sEOAD=sporadic early-onset Alzheimer disease.

a ANOVA *P* = .002; Tukey *P* < .05 for sEOAD vs both *PSEN1* groups.

b ANOVA *P* = .022; Tukey *P* < .05 for sEOAD vs *PSEN1*-Col group.

c ANOVA *P* = .012; Tukey *P* < .05 for sEOAD vs *PSEN1* Col group.

d ANOVA *P* = .0001; Tukey *P* < .05 for sEOAD vs both *PSEN1* groups.

*ApoE*-ε4 and *apoE*-ε2 = number and percentage of cases with one or more *ApoE* ε-4 or ε-2 alleles. Means and medians are shown for the following: NPTHAL, Thal amyloid phase; NPBRAAK, Braak neurofibrillary stage; NPNEUR, CERAD neuritic plaque density; NPDIFF, diffuse plaque density; NPADNC, NIA-AA AD Neuropathological Change level; NPAMY, cerebral amyloid angiopathy density.

Amyloid angiopathy, which is very consistently present in AD, was the only AD-associated pathology type with widely differing severity scores between the 3 groups, with median scores of 3, 2, and 1 in the *PSEN1* Colombia, *PSEN1* United States and sEOAD cases, respectively (Table 2). ANOVA showed very significant group differences as well as significant pairwise differences between both *PSEN1* groups and the sEOAD group.

Apolipoprotein E (*APOE*) genotype, which strongly influences not only the prevalence and age of onset of AD but also the prevalence of amyloid angiopathy, did not show significant proportional group differences for the possession of an ε4 or ε2 allele, although the United States *PSEN1* and sEOAD groups had 1.6- and 1.8-fold greater ε4 possession as compared to the Colombian *PSEN1* group (Table 2). Possession of the ε2 genotype was relatively rare among all groups, between ∼3% and 14%.

The proportion of cases lacking any typical AD comorbidities, classified here as “AD-Only” cases (Table 3), was low for both the *PSEN1* groups, less than 30% and only a little higher, 44%, for the sEOAD group. The difference in proportions between groups was not significant.

**Table 3.** Autopsy data for sEOAD and *PSEN1* cases, showing proportions of common AD comorbidities.

Group	AD Only	NPLBOD	NPHIPSCL	TDPNOS	NPFTDTAU
***PSEN1*—United States**	2/19; 10.5%	10/19; 52.6%	0/19; 0%	3/19; 15.8%	0/19; 0%
***PSEN1*—COL**	5/17; 29.4%	12/17; 70.6%	0/50; 0%	3/17; 17.6%	0/36; 0%
**sEOAD**	8/18; 44.4%	19/33; 57.6%	1/32; 3.1%	2/20; 10.0%	1/33; 3.0%

Abbreviations: *PSEN1*=presenilin-1; sEOAD=sporadic early-onset Alzheimer disease.

Numerator is the number of cases meeting criteria for the comorbidity while denominator is the number of ADD cases evaluated for the condition; also given is percentage. AD Only, AD without any of the other conditions in this table and without NPINF, gross infarcts including lacunes; NPOLD, old microinfarcts; NPOLDD, old cerebral microhemorrhages; NPLBOD, Lewy body disease; NPHIPSCL, hippocampal sclerosis; TDPNOS, TDP-43 pathology in amygdala, hippocampus, or entorhinal area; NPFTDTAU, non-AD tau pathology (PSP, CBD, Pick’s, argyrophilic grains, other).

Of AD comorbidities, LBD was most common (Table 3), being present in more than half of all cases in this study, and was particularly common in the Colombian *PSEN1* and sEOAD cases, at approximately 70% for each group; the differences in group proportions were not significant, however. Of those cases with LBD, the brain distribution in the majority of all groups was limbic predominant and amygdala-only, especially in the *PSEN1* groups (Table 4). The brainstem-predominant stage was uncommon in all groups. The olfactory bulb-only stage was not seen in the sporadic group but was significantly more common, at 30%, in the United States *PSEN1* group, as compared to 8% for the Colombian *PSEN1* group. The neocortical stage was not seen in the United States *PSEN1* group and was only about 10%-12% of the sEOAD and Colombian groups; these differences were not significant.

**Table 4.** Autopsy data for sEOAD and *PSEN1* cases, showing brain stages of cases with Lewy body disease.

Group	**LB-OB** ^a^	LB-BS	LB-Amyg	LB-Limb	LB-Neo
***PSEN1*—United States**	3/10; 30%	0/10; 0%	3/10; 30%	4/10; 40%	0/10; 0%
***PSEN1*—COL**	1/12; 8.3%	1/12; 8.3%	8/12; 66.7%	4/12; 26.7%	2/12; 11.8%
**sEOAD**	0/33; 0%	1/33; 3.0%	10/33; 30.3%	4/33; 12.1%	3/33; 10.0%

Abbreviations: LB-OB=olfactory bulb only; LB-BS=brainstem predominant; LB-Amyg=amygdala predominant; LB-Limb=limbic (transitional); LB-Neo=Neocortical (diffuse); *PSEN1*=presenilin-1; sEOAD=sporadic early-onset Alzheimer disease.

a Fisher exact test *P* = .002 for United States *PSEN1* vs *PSEN1* Col group.

Numerator is the number of cases meeting criteria for the stage while denominator is the number of ADD cases that were positive in any region for Lewy body disease; also given is percentage.

For TDP-43 co-pathology (Tables 3 and 5), the prevalences were not significantly different between groups, at 17.6%, 15.8%, and 10%, for the Colombian *PSEN1* group, United States *PSEN1* group, and sEOAD cases, respectively. For all United States *PSEN1* and sEOAD cases, TDP-43 pathology was confined to the amygdala and/or hippocampus and/or entorhinal area without any pathology in the neocortex or spinal cord. Three Colombian *PSEN1* cases (out of 17 stained) were positive in the amygdala, with one of these also positive in the hippocampus; the entorhinal area was not stained and there was no neocortical or spinal cord positive staining. Notably, hippocampal sclerosis and non-AD tau pathological conditions were not present in any of the United States or Colombian *PSEN1* cases and were seen in only 3% of the sEOAD cases.

**Table 5.** Autopsy data for sEOAD and *PSEN1* cases, showing brain regional positivity for TDP-43 proteinopathy.

Group	NPTDPB	NPTDPC	NPTDPD	NPTDPE	NPTDPA
	Amyg	Hip	Ent	Neocx	SpCd
***PSEN1*—United States**	1/18; 5.0%	3/19; 15.8%	3/19; 15.8%	0/18; 0%	0/12; 0%
***PSEN1*—COL**	3/17; 17.6%	1/17; 5.88%	0/3; 0%	0/17; 0%	0/17; 0%
**Sporadic—United States**	2/18; 11.1%	1/17; 5.88%	1/20; 5.0%	0/14; 0%	0/6; 0%

Abbreviations: LB-OB=olfactory bulb only; LB-BS = brainstem predominant; Amyg = amygdala; Hip = hippocampus; Ent = entorhinal; predominant; Neocx = neocortex; SpCd = spinal cord; *PSEN1* = presenilin-1; sEOAD = sporadic early-onset Alzheimer disease.

Numerator is the number of cases that were positive while denominator is the number of cases that were evaluated in each region; also given is percentage that were positive.

Cerebrovascular disease categories varied between the sEOAD and *PSEN1* groups (Table 6). Significant large-vessel atherosclerosis, assessed by NACC as a circle of Willis atherosclerosis (NACCAVAS), was present in a much larger percentage of Colombian *PSEN1* cases, at almost 19% as compared to 0% and 6% of the United States *PSEN1* and sEOAD cases, respectively. The group differences were significant as was the comparison between sEOAD cases vs Colombian *PSEN1* cases. Small-vessel disease, or arteriolosclerosis (NACCARTE) was much more common than large vessel disease, being present in all groups between 18% and 37%; the group differences were not significant on a Chi-square test. The consequences of vascular disease include gross and microscopic infarcts, as well as microscopic hemorrhages, but all were generally absent or present at very low percentages in all groups.

**Table 6.** Autopsy data for sporadic sEOAD and *PSEN1* cases, listing major cerebrovascular comorbidities.

Group	**NACCAVAS** ^a^	NPINF	NPOLD	NPOLDD	NACCARTE	**NPWMR** ^b^
***PSEN1*—United States**	0/19; 0%	2/19; 10.5%	0/19; 0%	0/19; 0%	7/19; 36.8%	11/19; 57.9%
***PSEN1*—COL**	9/48; 18.75%	0/25; 0%	2/48; 4.2%	1/48; 2.1%	9/36; 25%	N/A
**sEOAD**	2/33; 6.1%	0/33; 0%	2/33; 6.1%	0/29; 0%	6/32; 18.7%	5/28; 17.9%

Abbreviations: NACCAVAS=severity of atherosclerosis of circle of Willis>mild; NPINF=gross infarcts including lacunes; NPOLD = old microinfarcts; NPOLDD = old cerebral microhemorrhages; NACCARTE = arteriolosclerosis>mild; NPWMR = white matter rarefaction>mild; *PSEN1* = presenilin-1; sEOAD = sporadic early-onset Alzheimer disease.

a Chi-square test *P* = .05 between groups; Fisher exact test *P* = .04 for sporadic vs *PSEN1*—COL.

b Fisher exact test *P* = .01 between EOADD and United States *PSEN1* groups.

The numerator is the number of cases meeting criteria for the condition, while the denominator is the number of cases evaluated for the condition.

White matter rarefaction (NPWMR, Table 6), conventionally regarded as a consequence of arteriolosclerosis, was remarkably common, at almost 60%, in the United States *PSEN1* group, as compared to about 18% in the sEOAD cases, a significant difference. WMR was not assessed in the Colombian *PSEN1* cases.

To investigate whether AD comorbidities have a significant clinical effect in these early-onset cases, we used logistic regression models with age of onset and disease duration as the clinical dependent variables (Tables 7 and 8). We also sought to determine the predictive variables for the prevalence of CAA (NPAMY) or circle of Willis atherosclerosis (AVAS), as both of these pathologies are enriched in those with a *PSEN1* mutation. To obtain sufficient statistical power, all groups were included in the models. As there is an unequal distribution of female sex, race, and Hispanic ethnicity among the groups in this study, in particular with *PSEN1* cases being twice as likely to be female and sEOAD cases 3 times as likely to be male, variables in the models also included female sex, race, and Hispanic ethnicity. Stepwise elimination of non-significant variables indicated 3 attributes that predicted an earlier onset age: possession of a *PSEN1* mutation, possession of an *APOE*- ε4 genotype, and presence of TDP-43 proteinopathy (Table 7). A shorter disease duration was predicted only by the absence of a *PSEN1* mutation, as sEOAD cases had significantly shorter disease durations. This result may have been biased, however, by the experimental design that excluded sporadic AD cases that died older than age 60 years. Variables found to be non-significant predictors included sex, race, circle of Willis atherosclerosis, LBD (separate analyses were done for any LBD vs only limbic-transitional-neocortical stages), gross infarcts, microscopic infarcts, microscopic hemorrhages, arteriolosclerosis, and cerebral WMR.

**Table 7.** Multivariable logistic regression models for the effects of independent variables on earlier age of onset.

Independent variable	Beta coefficient	Standard error	Wald coefficient/standard error	Probability	OR	95% CIs
*PSEN1* mutation	2.25718	.629793	3.58401	.000338	9.556	2.781-32.838
ApoE-ε4	.011939	.006192	1.92814	.053837	1.012	1.012-1.024
TDP-43	.020351	.005728	3.55281	.000381	1.021	1.009-1.032

Abbreviation: *PSEN1* = presenilin-1.

The significant or near-significant independent variables are presence or absence of a *PSEN1* mutation, possession of an apolipoprotein E -ε4 allele and presence of TDP-43 pathology. Only results for significant or near-significant predictor variables are shown.

**Table 8.** Multivariable logistic regression models for the effects of independent variables on disease duration.

Independent variable	Beta coefficient	Standard error	Wald coefficient/standard error	Probability	OR	95% CIs
*PSEN1* mutation	2.25767	.629884	3.58427	.000338	9.561	2.782-32.86

Abbreviations: *PSEN1*=presenilin-1; sEOAD = sporadic early-onset Alzheimer disease.

The only significant independent variable is presence or absence of a *PSEN1* mutation; sEOAD subjects had significantly shorter disease durations. Only results for significant variables are shown.

For CAA (Table 9), the only significant predictors were Hispanic ethnicity (strong positive predictor) and white race (strong negative predictor). For circle of Willis atherosclerosis severity, all models failed due to multicolinearity between race, Hispanic ethnicity, and sex. This was expected due to the preponderance of a threshold severity of circle of Willis atherosclerosis (greater than mild) in Colombian, Hispanic, multiracial females, ie, in 9/11 of those meeting that threshold.

**Table 9.** Multivariable logistic regression models for the effects of independent variables on the likelihood of severe CAA (NPAMY score greater than mild).

Independent variable	Beta coefficient	Standard error	Wald coefficient/standard error	Probability	OR	95% CIs
Hispanic	1.05470	.269112	3.91918	.000089	2.871	1.694-4.865
White	−1.00476	.268312	−3.74473	.000181	.3661	.216-.366

The only significant independent variables are Hispanic ethnicity (a positive predictor) and White race (a negative predictor). Only results for significant variables are shown.

## DISCUSSION

This report updates an earlier comparison by Ringman et al^10^ of NACC-classified neuropathology for *PSEN1* and sEOAD cases, based on the prior NP9 NACC neuropathology data elements. We used accumulated data from the NP10 version, which is more extensive in its inclusion of AD comorbidities. Additionally, we included a comparison with *PSEN1* E280A cases from the Colombian kindred, which have not previously been systematically evaluated for AD comorbidities.

Confirmatory of earlier NACC-based reports,^10^^,^^21^ we find that the mean severity scores for amyloid plaques and CAA are uniformly greater in *PSEN1* AD cases as compared with those for sporadic AD, but in this study, we have also found that sporadic AD cases dying before age 60 years have amyloid plaque pathology that is almost equivalent in severity to that found in *PSEN1* mutation cases. This concurs with previous reports of sporadic AD histopathology^6^^,^^7^ documenting the greatest severity in the cases with the earliest onset, as well as progressively decreased severity with advancing age afterward.

The *APOE-ε*4 allele generally confers an earlier age of onset and greater clinical and neuropathological severity of AD. The background prevalence of an *ε*4 allele in the “Paisa” population of Colombia, which includes the E280A mutation carriers included in this study, is 11%,^22^ while its population frequency in a pooled analysis of European-derived subjects has been reported as being between 7.5% and 15.6%.^23^ These frequencies are considerably higher, averaging around 40%, in subjects with AD dementia,^24^ similar to the prevalences found in the present study, ranging from 26.5% to 48.5% but with non-significant differences between groups.

Cerebral amyloid angiopathy (CAA) was the only AD-associated pathology type with markedly differing severity scores between the 3 studied groups, with median scores of 3, 2, and 1 in the *PSEN1* Colombia, *PSEN1* United States and sEOAD cases, respectively. The greater CAA severity in *PSEN1* mutation-associated AD has been previously documented^10^ and is of particular relevance because of the greater risk with CAA for amyloid-related imaging abnormalities, vasogenic edema, and hemorrhage, which are limiting factors for anti-amyloid monoclonal antibody therapy. Other predisposing factors to CAA include the *APOE*-ε4 allele, hypercholesterolemia, and Hispanic ethnicity,^25^^,^^26^ and, within *PSEN1* cases, mutations beyond codon 200.^10^ Our logistic regression model confirmed Hispanic ethnicity as a significant independent predictor of CAA severity. The greater prevalence of higher severity CAA in the E280A Colombian cases might therefore be at least partially due to the higher-codon mutation location, greater proportion with Hispanic ethnicity and greater likelihood of hypercholesterolemia in Colombia as compared to the United States,^27^ although the current set of Colombian subjects had a lower *APOE*- ε4 carriage rate. Despite the severe CAA severity, only 1/67 combined *PSEN1* cases, and no sEOAD cases, had gross or microscopic brain hemorrhages.

The distribution of *APOE-*ε4 and *APOE*- ε2 alleles differed between the studied groups. The *APOE*-ε4 allele was most common in the *PSEN1* United States group, followed by the sEOAD group. Carriage of an *APOE-*ε4 or ε2 allele has been associated with earlier or later clinical onset, respectively, in both sporadic AD and *PSEN1* AD subjects^28^^,^^29^; our logistic regression analysis confirmed this effect of the *APOE-*ε4 allele but we had insufficient numbers of *APOE-*ε2 subjects to confirm the earlier reports. The United States *PSEN1* group had a higher proportion of *APOE-*ε4 carriers than the Colombian *PSEN1* group, perhaps contributing to their earlier mean onset age in the current study subjects. There was an extremely low proportion (1/34) of Colombian *PSEN1* E280A cases with the *APOE-*ε2 allele but with this small sample, this was not significantly different from the other groups. A single case of the Christchurch *APOE-*ε3 mutation, which in some respects has biochemical similarities with the *APOE-*ε2 allele, has recently been reported in the Colombian *PSEN1* kindred^30^; this case had a remarkable 30-year delay of clinical onset as well as reduced levels of ADNC.^31^ Other disease-modifying genetic loci have been described for *PSEN1* AD^10^^,^^28^^,^^32–34^ and these offer important clues for the design of disease-modifying strategies.

AD comorbidities are not restricted to the oldest-old^6^^,^^7^ as some comorbidity types are common even in early-onset AD, whether sporadic or inherited. The percentage of cases with ADNC as the sole pathology, defined as the absence of LBD, TDP-43, non-AD tauopathy, infarcts, and microhemorrhages, was low in all groups. The NACC *PSEN1* cases had the lowest rate of AD as a sole major pathology, at ∼10% while this rate was ∼29% for the Colombian *PSEN1* E280A cases and 48% for the sEOAD group.

Of typical AD comorbidities, LBD is the most common in subjects under age 80 years,^6^ and reaches very high prevalences in this study’s *PSEN1* and sEOAD subjects. Colombian *PSEN1* cases had the highest LBD comorbidity rates, at ∼70%, as compared to the United States sEOAD and *PSEN1* groups, at 57% and 53%, respectively. The rates we report here for the *PSEN1* and United States sEOAD cases are considerably higher than the approximately 30% prevalences reported by Ringman et al^10^; this may be due to interim improvements in the immunohistochemical detection of pathological α-synuclein, and to the addition, in the NACC NP10 dataset, of the olfactory bulb-only LBD stage. In another, more recent study of 12 *PSEN1* United States cases,^12^ 6 (50%) had comorbid α-synuclein pathology.

As the gradual decline in LBD prevalence with age in sporadic AD closely parallels the decline in AD pathology severity with age,^6^ but surprisingly reaches very high prevalences in these *PSEN1* and EOAD cases, this might suggest that LBD in this setting is largely dependent on AD pathology, rather than age. The high reported rates of LBD in not only *PSEN1* AD but also in other forms of autosomal dominant AD,^35–41^ Down’s syndrome, and even in other, non-Aβ cerebral amyloidosis,^42–45^ supports this conclusion. Our logistic regression models, however, did not find LBD to be a significant predictor of an earlier symptomatic onset or of a shorter disease duration.

Comorbid TDP-43 proteinopathy has previously been investigated in human *PSEN1* AD but not in *PSEN1* with the E280A mutation. Two separate United States studies of a combined 42 cases both reported limbic-restricted TDP-43 pathology in 17% of subjects with varying *PSEN1* genotypes.^42^^,^^43^ We confirmed this finding, as TDP-43 pathology affected 15%-17% of the *PSEN1* cases studied here. The sEOAD cases had a somewhat lower prevalence, at 11%. Concurrent TDP-43 proteinopathy is also found in 14% of Down’s syndrome cases,^42^ further directly implicating AD pathology. We found that the presence of TDP-43 pathology, together with *PSEN1* and *APOE-*ε4 genotypes, was predictive of an earlier age of onset. Whether TDP-43 pathology is a cause or an effect of earlier onset in these subjects is not determined by these results. To our knowledge, at least in sEOAD, TDP-43 pathology has been associated with older, rather than younger age.^6^

Hippocampal sclerosis, however, which in older people is often associated with hippocampal TDP-43 pathology,^46^ was present in only one sEOAD case and in none of the United States or Colombian *PSEN1* cases; it would therefore seem to require aging as a co-factor. It is likely that non-AD tauopathies, which were rare in these younger subjects, are not common or direct consequences of ADNC and probably are more age-related, as has been shown for sporadic AD cases over a wide age range.^6^^,^^7^

Significant large-vessel atherosclerosis (AVAS), which is usually regarded as age-dependent, was present in a surprisingly large percentage of Colombian *PSEN1* cases, at almost 20% as compared to 0% and 3% of the United States *PSEN1* and sporadic United States cases, respectively. We investigated, with logistic regression, whether or not the almost-exclusively Colombian female sex distribution of severe AVAS might account for the greater AVAS severity that we found in the Colombian group. Generally, men are considered to be at higher risk for significant cerebral atherosclerosis but Hispanic ethnicity is also a risk factor for intracranial atherosclerotic stenoses.^47^^,^^48^ Whether this concentration of AVAS severity in Colombian women with a *PSEN1* mutation represents more than just a statistical anomaly is uncertain but may be less related to the E280A mutation or AD pathology than to the generally higher cardiovascular risk factors in Colombia as compared to the United States.^27^

Small-vessel disease, or arteriolosclerosis, was more common than large-vessel disease in all 3 of the studied groups, with ascending rates between 18% and 37% going from sporadic to Colombian to United States *PSEN1* groups. Arteriolosclerosis has been reported to be unrelated to CAA in the *PSEN1* Colombian kindred.^16^ Intriguingly, the most severe forms of arteriolosclerosis are found in CADASIL, an autosomal dominant condition^49^ caused by Notch-3 mutations, while *PSEN1* acts enzymatically on all Notch gene products.^50^

The consequences of cerebrovascular disease include gross and microscopic infarcts, as well as gross and microscopic hemorrhages; all were generally absent or rare in all groups, however. As mentioned, it might have been expected that microhemorrhages would be much more common in *PSEN1* AD due to the high rates of CAA. However, these may be underestimated at autopsy as compared to MRI, or when special efforts are made in postmortem examination.^51^ In the Alzheimer’s Disease Neuroimaging Initiative, 25% of a mixed group of subjects, with normal cognition, mild cognitive impairment, and AD dementia, had microhemorrhages, with a tendency for proportionately more in AD.^52^

WMR, conventionally regarded as a consequence of arteriolosclerosis, was remarkably common, at almost 60%, in the United States *PSEN1* group, as compared to 18% in the sEOAD cases. We did not have postmortem estimates of WMR for the Colombian E280A group but previous MR reports^53^^,^^54^ found increased mean diffusivity and white matter hyperintensity volumes compared to age-similar healthy controls. WMR has long been noticed to be roughly twice as common in AD as compared to normal elderly,^55^^,^^56^ and is reportedly pronounced relative to age-matched controls in subjects with *PSEN1* and *APP* mutations and Down syndrome.^57–62^ The greater WMR in the United States *PSEN1* group studied here, relative to the sEOAD group, may be in part due to the higher proportion of significant arteriolosclerosis, 37% vs 18%, in the *PSEN1* group as compared to the sEOAD group, but could also be due to the more severe AD histopathological scores as WMR probably has a dual vascular and AD-related pathogenesis.^63–65^ Like LBD, WMR is highly prevalent in autosomal dominant AD and thus this adds to other evidence that it is at least partially dependent on ADNC. Both arteriolosclerosis and WMR rarefaction were most common in the United States *PSEN1* cases.

Effective clinical trials depend on accurate estimates of required subject numbers, which largely depend on accurate assumptions for effect size and variability. Variability of clinical rates of decline will require larger subject numbers to overcome. Effect sizes depend on the efficacy of the therapeutic agent, which may be dependent on a matching of molecular mechanisms of agent and pathogenesis. Neuropathological comorbidities in AD may all affect cognition but may have completely different molecular pathogenesis and therefore might not respond to ADNC-specific therapeutics, leading to loss of efficacy, effect size, and probability of clinical trial success. Stratifying AD subjects by the presence and types of accompanying comorbidities might result in increased observed effect sizes in some groups as compared to others, potentially “rescuing” failed clinical trials.

An important limitation of this study is the relatively small subject numbers, particularly for the United States *PSEN1* group. Some statistical associations may therefore be spurious or undetected. Additionally, the neuropathology assessment may have differed between centers, possibly accounting for some of the group differences that we have observed.

This study is the first to compare rates of AD comorbidities in the *PSEN1* Colombian E280A kindred with those in other *PSEN1* mutation types as well as with similarly aged sporadic AD cases. The results presented here, as well as other evidence, indicate that LBD, TDP-43 pathology, and WMR, as common comorbidities with autosomal dominant and early-onset sporadic AD, should be considered when planning clinical trials with such subjects as they may increase variability in response rates. However, individual genetic variability beyond possession of a *PSEN1* mutation will undoubtedly be influential and future studies should examine this. To some extent, though, this study suggests that AD comorbidities may be at least partially dependent on ADNC and thus potentially addressable by anti-amyloid or anti-tau therapies.

## Supplementary Material

nlae122_Supplementary_Data
