# Correction to: Comorbidities in early-onset sporadic versus presenilin-1 mutation-associated Alzheimer disease dementia: Evidence for dependency on Alzheimer disease neuropathological changes

**DOI:** 10.1093/jnen/nlaf030

**Published:** 2025-04-18

This is a correction to: Diego Sepulveda-Falla, Carlos Andrés Villegas Lanau, Charles White III, Geidy E Serrano, Juliana Acosta-Uribe, Barbara Mejía-Cupajita, Nelson David Villalba-Moreno, Pinzhang Lu, Markus Glatzel, Julia K Kofler, Bernardino Ghetti, Matthew P Frosch, Francisco Lopera Restrepo, Kenneth S Kosik, Thomas G Beach, Comorbidities in early-onset sporadic versus presenilin-1 mutation-associated Alzheimer disease dementia: Evidence for dependency on Alzheimer disease neuropathological changes, *Journal of Neuropathology & Experimental Neurology*, Volume 84, Issue 2, February 2025, Pages 104–113, https://doi.org/10.1093/jnen/nlae122

In the originally published version of the manuscript, the academic degrees for authors Mathew P. Frosch, Markus Glatzel, and Kenneth S. Kosik were incorrectly indicated. The correct listings are Matthew P. Frosch, MD, PhD, Markus Glatzel, MD, and Kenneth S. Kosik, MD.

The emendations have been made to the article.

