# Supplementary material for: Comorbidities in early-onset sporadic versus presenilin-1 mutation-associated Alzheimer disease dementia: Evidence for dependency on Alzheimer disease neuropathological changes
Source: J Neuropathol Exp Neurol. 2024 Dec 4;84(2):104–13. doi: 10.1093/jnen/nlae122 (PMC11747142; doi:10.1093/jnen/nlae122)
Supplement: nlae122_Supplementary_Data [file nlae122_supplementary_data.docx]

**Table 1.**  Demographics and mutation genotypes for *PSEN1* and sporadic EOAD (sEOAD) cases.

| **Group** | **Age at Onset^1^**  **(mean, SD, range)** | **A. Age at Death^2^**  **B. Disease**  **Duration^3^**  **(mean, SD, range)** | **Sex** | ***PSEN1* Mutation Genotypes** | |
| --- | --- | --- | --- | --- | --- |
|  |  |  |  | **Mutation** | **N (Cases)** |
| ***PSEN1* - USA**  **N = 19** | 42.81; 6.66; 33-58 | A. 53.4; 8.6; 34-75  B. 11.6; 5.12; 603 | 6 M, 13F | A431E  G206A  A426P  P88L  H163R  P267A  G206E  M233L  N135S  S130L | 5  3  2  2  2  1  1  1  1  1 |
| ***PSEN1* - Colombia**  **N = 50** | 47.8; 5.32; 37-62 | A.57.7; 7.25; 42-79  B. 9.88; 4.52; 2-22 | 15M, 35F | E280A12 (All) | |
| **Sporadic - USA**  **N = 33** | 48.3; 2.84; 41-54 | A. 56.3; 2.98; 47-59  B. 4.0; 1.87; 1-10 | 25M; 8 F | N/A | |
| **Sporadic – USA**  **Confirmed Non-Genetic**  **N = 18** | 48.2; 2.39; 43-52 | A. 56.4; 2.28; 52-59  B. 3.94; 2.07; 2-10 | 14M; 4 F | N/A | |

^1^ANOVA p = 0.003; Tukey p < 0.01 for *PSEN1-USA* vs all other groups.

^2^ANOVA p = 0.075; Tukey p < 0.05 for *PSEN1-USA* vs *PSEN1-COL.*

^3^ANOVA p < 0.0001; Tukey p < 0.001 for *PSEN1 groups vs both* Sporadic-USA groups*.*

**Table 2.** Autopsy data for AD-related neuropathological variables in sEOAD and *PSEN1* AD cases. *ApoE*-ε4 and *apoE*-ε2 = number and percentage of cases with one or more *ApoE* ε-4 or ε-2 alleles. Means and medians are shown for the following: NPTHAL = Thal amyloid phase; NPBRAAK = Braak neurofibrillary stage; NPNEUR = CERAD neuritic plaque density; NPDIFF = diffuse plaque density; NPAMY = cerebral amyloid angiopathy density; NPADNC = NIA-AA AD Neuropathological Change level.

| **Group** | **ApoE-4** | **ApoE-2** | **NPTHAL^1^** | **NPBRAAK** | **NPNEUR** | **NPDIFF^2^** | **NPADNC^3^** | **NPAMY^4^** |
| --- | --- | --- | --- | --- | --- | --- | --- | --- |
| ***PSEN1*-US** | 6/14  42.9% | 2/14  14.3% | 5; 5 | 6; 6 | 3; 3 | 3; 3 | 3; 3 | 2.10; 2 |
| ***PSEN1*-COL** | 9/34  26.5% | 1/34  2.94% | 5; 5 | 6; 6 | 2.9; 3 | 3; 3  (n = 36) | 3; 3 | 2.58; 3 |
| **sEOAD (all)** | 16/33  48.5% | 2/33  6.1% | 4.72; 5 | 5.79; 6 | 2.85; 3 | 2.70; 3 | 2.88; 3 | 1.42; 1 |
| **sEOAD Confirmed Non-Genetic** | 10/18  55.5% | 2/18  11.1% | 4.67; 5 | 5.83; 6 | 2.89; 3 | 2.78; 3 | 2.78; 3 | 1.22; 1 |

^1^ANOVA p = 0.002; Tukey p < 0.05 for SEOAD vs both *PSEN1* groups.

^2^ANOVA p = 0.022; Tukey p < 0.05 for SEOAD vs *PSEN1*-Col group.

^3^ANOVA p = 0.012; Tukey p < 0.05 for SEOAD vs *PSEN1* Col group.

^4^ANOVA p = 0.0001; Tukey p < 0.05 for SEOAD vs both *PSEN1* groups.

**Table 3.**  Autopsy data for sEOAD and *PSEN1* cases, showing proportions of common AD comorbidities. Numerator is the number of cases meeting criteria for the comorbidity while denominator is the number of AD cases evaluated for the condition; also given is percentage. AD Only = AD without any of the other conditions in this table and without NPINF, NPOLD and NPOLDD from Table 5; NPLBOD = Lewy body disease; NPHIPSCL = hippocampal sclerosis; TDPNOS = TDP-43 pathology in amygdala, hippocampus, entorhinal, neocortex or spinal cord; NPFTDTAU = non-AD tau pathology (PSP, CBD, Pick’s, argyrophilic grains, other).

| **Group** | **AD Only** | **NPLBOD** | **NPHIPSCL** | **TDPNOS** | **NPFTDTAU** |
| --- | --- | --- | --- | --- | --- |
| ***PSEN1*-US** | 2/19; 10.5% | 10/19; 52.6% | 0/19; 0% | 3/19; 15.8% | 0/19; 0% |
| ***PSEN1*-COL** | 5/17; 29.4% | 12/17; 70.6% | 0/50; 0% | 3/17; 17.6% | 0/36; 0% |
| **sEOAD (all)** | 8/18; 44.4% | 19/33; 57.6% | 1/32; 3.1% | 2/20; 10.0% | 1/33; 3.0% |
| **sEOAD Confirmed Non-Genetic** | 2/5; 40% | 7/18; 38.9% | 1/17; 5.8% | 0/12; 0% | 1/18; 5.5% |

**Table 4.**  Autopsy data for sEOAD and *PSEN1* cases, showing brain stages of cases with Lewy body disease. Numerator is the number of cases meeting criteria for the stage while denominator is the number of AD cases that were positive in any region for Lewy body disease; also given is percentage. LB-OB = olfactory bulb only; LB-BS = brainstem predominant; LB-Amyg = amygdala predominant; LB-Limb = limbic (transitional); LB-Neo = Neocortical (diffuse).

| **Group** | **LB-OB^1^** | **LB-BS** | **LB-Amyg** | **LB-Limb** | **LB-Neo** |
| --- | --- | --- | --- | --- | --- |
| ***PSEN1*-US** | 3/10; 30% | 0/10; 0% | 3/10; 30% | 4/10; 40% | 0/10; 0% |
| ***PSEN1*-COL** | 1/12; 8.3% | 1/12; 8.3% | 8/12; 66.7% | 4/12; 26.7% | 2/12; 11.8% |
| **sEOAD (all)** | 0/33; 0% | 1/33; 3.0% | 10/33; 30.3% | 4/33; 12.1% | 3/33; 10.0% |
| **sEOAD Confirmed Non-Genetic** | 0/18; 0% | 0/18; 0% | 5/18; 27.8% | 1/18; 5.5% | 1/18; 5.5% |

^1^Fisher exact test p = 0.002 for US *PSEN1* vs *PSEN1* Col group.

**Table 5.** Autopsy data for sEOAD and *PSEN1* cases, listing major cerebrovascular comorbidities. The numerator is the number of cases meeting criteria for the condition, while the denominator is the number of cases evaluated for the condition. NACCAVAS = severity of atherosclerosis of circle of Willis > mild; NPINF = gross infarcts including lacunes; NPOLD = old microinfarcts; NPOLDD = old cerebral microhemorrhages; NACCARTE = arteriolosclerosis > mild; NPWMR = white matter rarefaction > mild.

| **Group** | **NACCAVAS^1^** | **NPINF** | **NPOLD** | **NPOLDD** | **NACCARTE** | **NPWMR^2^** |
| --- | --- | --- | --- | --- | --- | --- |
| ***PSEN1*-US** | 0/19; 0% | 2/19; 10.5% | 0/19; 0% | 0/19; 0% | 7/19; 36.8% | 11/19; 57.9% |
| ***PSEN1*-COL** | 9/48; 18.75% | 0/25; 0% | 2/48; 4.2% | 1/48; 2.1% | 9/36; 25% | N/A |
| **sEOAD (all)** | 1/33; 3.0% | 0/33; 0% | 2/33; 6.1% | 0/29; 0% | 6/32; 18.7% | 5/28; 17.9% |
| **sEOAD Confirmed Non-Genetic** | 1/18; 5.5% | 0/18; 0% | 1/18; 5.5% | 1/14; 0% | 3/18; 16.7% | 1/13; 7.7% |

^1^Chi-square test p = 0.05 between groups; Fisher exact test p = 0.04 for sporadic vs *PSEN1*-COL.

^2^Fisher exact test p = 0.01 between EOADD and US *PSEN1* groups.
